# Overexpression of Recombinant Human Beta Interferon (rhINF-β) in Periplasmic Space of Escherichia coli 

**Published:** 2014

**Authors:** Mohammad Hossein Morowvat, Valiollah Babaeipour, Hamid Rajabi-Memari, Hossein Vahidi, Nader Maghsoudi

**Affiliations:** a *Department of Pharmaceutical Biotechnology, School of Pharmacy, Shahid Beheshti University of Medical Sciences, Tehran, Iran.*; b *Biochemical Engineering Group, Biotechnology Research Center, Malek-Ashtar University of Technology, Tehran, Iran.*; c *Center of Biotechnology and Life sciences, Shahid Chamran University of Ahvaz, Iran.*; d *Department of Bioscience Engineering, Faculty of New Sciences and Technologies, University of Tehran, Tehran, Iran.*; e *Neuroscience Research Center, Shahid Beheshti University of Medical Science, Tehran, Iran. *

**Keywords:** Periplasmic expression, *Escherichia coli*, Interferon beta, Plasmid, Expression system

## Abstract

Human Interferon β (INF-β) is a member of cytokines family which different studies have shown its immunomodulatory and antiviral activities. In this study an expression vector was designed and constructed for expression of human INF-β-1b either in shake flasks or bench top bioreactor. The designed vector was constructed based upon pET-25b(+) with T7 promoter. Recombinant human beta interferon (rhINF-β) was codon optimized and overexpressed as a soluble, N-terminal *pelB *fusion protein and secreted into the periplasmic space of *Escherichia coli *BL21 (DE3). The sugar, Isopropyl-β-D-thiogalactopyranoside (IPTG) was used as a chemical inducer for rhINF-β production in the shake flasks and bench top bioreactor. Timing of beta interferon expression was controlled by using the T7 promoter. The rhINF-β protein was extracted from periplasmic space by osmotic shock treatment and the expression of the beta interferon encoding gene in random selected transformants, was confirmed by western and dot blot methods. The maximum of product formation achieved at the OD_600nm _= 3.42 was found to be 35 % of the total protein content of the strain which translates to 0.32 g L-1. The constructed vector could efficiently overexpress the rhINF-β into the periplasmic space of *E. coli*. The obtained yield of the produced rhINF-β was more than previous reports. The system is easily adapted to include other vectors, tags or fusions and therefore has the potential to be broadly applicable to express other recombinant proteins.

## Introduction

Human Interferon β (INF-β) is a member of type I interferons (IFNs) which includes a large family of structurally related cytokines that mediate the early innate immune response to viral infections. INF-β which is originally called fibroblast interferon has 166 aminoacids and its encoding gene lacks introns and no reported polymorphisms (1, 2). It has been shown that INF-β has antiviral, antiproliferative and immunomodulatory properties (3, 4). It has been examined for the treatment of multiple sclerosis (5), arthritis (6), infectious disease (7), genital condylomata acuminata (8), malignant melanoma therapy (9) and other oncological indications (10). Moreover, it has been used in some in vitro studies (11). Comprising 79% (3.19 billion dollars) of the total sales (4.0 billion dollars), INF-β brands, indicated for multiple sclerosis, are the biggest seller in cytokines sector of biopharmaceuticals (12).

There are two different types of recombinant INF-β; INF-β-1a, the first type is glycosylated form which is produced in mammalian cells such as CHO and its aminoacid sequence is identical to that of human endogenous form (13). The second type is INF β-1b which has 165 aminoacids and its 17th aminoacid is different from the human natural INF-β (Cys → Ser) (14) and is produced in *Escherichia coli*, hence, it is not glycosylated (15). 

The vast majority of recombinant proteins have been produced in the gram negative bacterium *Escherichia coli *because of its ability to grow rapidly and at high density on inexpensive substrates, the profound genetic and physiological characterization, the large number of compatible tools available for biotechnology specially cloning vectors and host strains and the simple process scale up (16, 17). The ability of *Escherichia coli *to secrete proteins into the periplasm may be of great interest which has some advantages over intracellular production of recombinant proteins as inclusion bodies. It simplifies downstream processing, stability, protein purification and N-terminal processing, and there is less protein degradation, besides it provides an oxidizing environment to promote proper folding of the produced proteins (18).

There are some reports describing the construction of novel vectors to express human INF-β in *E. coli *(19-23). However, there is no report demonstrating periplasmic express human INF-β during high cell density cultivation of *E. coli*. Human INF-β produced in *E. coli *is toxic for the bacterial host (24, 25); hence, periplasmic production of this recombinant protein easily accumulates it in the periplasm to inhibit its lethal effects to the cell.

The aim of the present study was to construct an expression vector that allows stable and effective periplasmic expression of rhINF-β-1b sclerosis (5), arthritis (6), infectious disease (7), genital condylomata acuminata (8), malignant melanoma therapy (9) and other oncological indications (10). Moreover, it has been used in some in vitro studies (11). Comprising 79% (3.19 billion dollars) of the total sales (4.0 billion dollars), INF-β brands, indicated for multiple sclerosis, are the biggest seller in cytokines sector of biopharmaceuticals (12).

There are two different types of recombinant INF-β; INF-β-1a, the first type is glycosylated form which is produced in mammalian cells such as CHO and its aminoacid sequence is identical to that of human endogenous form (13). The second type is INF β-1b which has 165 aminoacids and its 17th aminoacid is different from the human natural INF-β (Cys → Ser) (14) and is produced in *Escherichia coli*, hence, it is not glycosylated (15). 

The vast majority of recombinant proteins have been produced in the gram negative bacterium *Escherichia coli *because of its ability to grow rapidly and at high density on inexpensive substrates, the profound genetic and physiological characterization, the large number of compatible tools available for biotechnology specially cloning vectors and host strains and the simple process scale up (16, 17). The ability of *Escherichia coli *to secrete proteins into the periplasm may be of great interest which has some advantages over intracellular production of recombinant proteins as inclusion bodies. It simplifies downstream processing, stability, protein purification and N-terminal processing, and there is less protein degradation, besides it provides an oxidizing environment to promote proper folding of the produced proteins (18).

There are some reports describing the construction of novel vectors to express human INF-β in *E. coli *(19-23). However, there is no report demonstrating periplasmic express human INF-β during high cell density cultivation of *E. coli*. Human INF-β produced in *E. coli *is toxic for the bacterial host (24, 25); hence, periplasmic production of this recombinant protein easily accumulates it in the periplasm to inhibit its lethal effects to the cell.

The aim of the present study was to construct an expression vector that allows stable and effective periplasmic expression of rhINF-β-1b either in shake flasks and bench top bioreactor using a pET derived expression system which provides a rapid and economical method for heterologous protein production. The experimental validation of the feasibility and the functionality of the constructed plasmid are also examined.

## Experimental

*Bacterial strain and plasmid*

*E. coli *BL21 F- *ompT hsd*SB (rB-mB-) *gal dcm *(DE3) (Invitrogen, CA, USA) was used as the host for synthesis of rhINF-β. An oligonucleotide was designed regarding *E. coli *codon usage; using vectorNTI version 11.0 (Invitrogen, CA, USA) (26) and synthesized. The in-house software optimizes a variety of parameters that are critical to the efficiency of gene expression, including codon usage bias, GC content, CpG dinucleotides content, mRNA secondary structure, cryptic splicing sites, premature polyA sites, internal Chi (crossover hot-spot instigator) sites and ribosomal binding sites, negative CpG islands, RNA instability motifs, AU-rich elements, repeat sequences (direct repeats, reverse repeats, and dyad repeats or palindromes), and restriction sites that may interfere with cloning procedure. It was inserted into the *Xho*I and *Msc*I cloning sites. The pET-25b(+) vector (Novagen, CA, USA) containing the requested insert including the optimized synthetic human INF-β-1b was purchased from Biomatik Corporation, ON, Canada. 

Briefly, the designed plasmid consists of the strong inducible T7 promoter under the control of the lac-operator sequence, the ORF for encoding gene of the requested protein, followed by the T7 transcription terminator transcription, a ColE1 origin of replication, the β-lactamase gene under its own promoter to confer resistance to ampicillin with the strong transcription terminator of phage λ.

The prokaryotic host cells were transformed using CaCl2 method with the constructed plasmid and transforming clones were selected on LB (Luria-Bertani) medium with 100 μg mL-1 ampicillin. Plasmids were prepared by the alkaline lysis method (27) and purified using plasmid kits from Vivantis (Selangor, Malaysia).

Cloning into plasmid vectors was performed by standard methods (28). The insertion was sequenced to confirm its integrity. The bacterial stocks were kept at −70 °C in 20 % (v/v) glycerol for long-term usage.

*Shake flask culture*

A frozen culture (1.5 mL) of recombinant transformed cells was inoculated into 50 mL of primary culture medium (sterile TB medium) containing 100 μg mL-1 of ampicillin in a 250 mL Erlenmeyer shake flask and incubated overnight in an NB-205V shaking incubator (N-BIOTEK, Bucheon-si, South Korea) at 160 rpm, 37 °C. Primary seed culture (1 mL) was used to inoculate 100 mL of secondary culture medium (sterile corresponding medium) in a 500 mL Erlenmeyer shake flask and grown for 8 h at 37 °C at 160 rpm. The cell growth was monitored during cultivation by optical density measurements at 600 nm using photometry method and the sampling was carried out every one hours.

*Inoculum preparation for fermentation*

The recombinant plasmid was transformed into *E. coli *strain BL21 (DE3). An individual colony was selected from LB agar plate and inoculated into 5 mL of terrific broth (TB) medium containing 100 μg mL-1 ampicillin. The 5 mL *E. coli *culture was incubated in shaking incubator overnight at 37 °C. One hundred μL of the overnight culture was inoculated into 200 mL TB medium containing 100 μg mL-1 of ampicillin. It was grown in shaking incubator at 37 °C and after reaching to OD600nm=0.7-1.0, then it was transferred to the bench-top bioreactor as seed culture.

*Batch-culture experiment*

The batch fermentation was carried out in a bench-top 5.0 L Minifors fermenter system (Infors HT, Basel, Switzerland) with a working volume of 2 L. The system was equipped with pH, temperature and pO2 probes and four peristaltic pumps for the addition of alkali, acid, nutrients, salts or antifoam solutions. All the fermentation parameters were registered using a computer connection to the fermenter, equipped with IRIS software. It could remotely control all the fermentation parameters, included pH, pO2, agitation speed (rpm) and aeration (L·min-1) and also was used for data storage.

The inlet airflow used was 1.0 vvm. The dissolved oxygen was monitored using a polarographic oxygen electrode (Mettler Toledo, Switzerland) and maintained above 30% saturation throughout the experiment. The pH was measured using a glass electrode and controlled at pH 7.0 using 1.0 M NaOH and 1.0 M HCl. The temperature and agitation were maintained at 37 °C and 500 rpm, respectively. The foam was controlled by the addition of a silicone-based anti-foaming reagent. Samples were taken at different times and analyzed for OD600nm, and rhINF-β production. TB medium was used for the fermentation study and the cell growth was monitored during cultivation by optical density measurements at 600 nm using photometry method.

*Induction and expression of rhINF-β*

The cells were induced at OD600nm of 1.0 with 0.2 mM IPTG (Fermentas, Vilnius, Lithuania). A part of the culture was used as negative control without adding IPTG (pre-induction). After 1-4 hours of additional growth at 37 °C, cells were harvested by centrifugation at 5000 g for 10 min at 4 °C.

*Preparation of periplasmic protein*

The pellet was thawed to room temperature and resuspended to 1 mL in a freshly prepared hypertonic solution of cell lysis buffer containing 20 % (w/v) sucrose, 30 mM Tris-Cl (pH 8.0) and 1 mM EDTA (pH 8.0). The mixture was stored on ice for 30 minutes at 4 °C. Cells were centrifuged at 5000 g for 10 min at 4 °C and the supernatant was collected. Cells were resuspended to 1 mL in a hypotonic solution of ice cold 5 mM MgSO4 and incubated for 30 min at 4 °C. The mixture was then centrifuged and the supernatant of hypotonic solution was collected and combined with the supernatant from the hypertonic solution, followed by an additional centrifugation to remove debris. The supernatant was collected immediately for the periplasmic proteins. The total soluble protein was analyzed by the Bradford method with bovine serum albumin (BSA) as a standard (29).

*Immunoblot analysis of the Recombinant hINF-β*

The resulting bacterial pellet or purified protein were homogenized in SDS sample loading buffer containing 0.150 M Tris–Cl, pH 6.8; 10 % glycerol; 2 % SDS; 0.01 % bromophenol blue; and 0.5 M 2-ME (2-Mercaptoethanol). It was mixed properly and boiled for 10 min and centrifuged at 11,000 g for 2 min. Electrophoresis was performed in the presence of SDS according to the method of Laemmli (30). The discontinuous gel consisted of a 4 % stacking gel and a 17.5 % separating gel was run on a vertical electrophoresis unit (omniPAGE mini, Cleaver Scientific, UK). Two commercial non-glycosylated rhINF-βs (Betaseron®, Bayer HealthCare, Germany and ZiferonTM, Zist Daru Danesh, Tehran, Iran) were used as the standard. Gels were stained with Coomassie Brilliant Blue R250. The total protein pattern of the recombinant bacteria, visualized on Coomassie brilliant blue stained gel, were scanned by a Bio-Rad Gel Doc 2000 densitometric gel scanner with an accuracy of greater than 95 %.

For western blotting experiment, electrophoresed proteins were transferred to a nitrocellulose membrane using a semi-dry method in a transfer buffer (25 mM Tris, 192 mM glycine, 20 % methanol) at 20 mA for 7 minutes using iBlot® western detection kit (Invitrogen, CA, USA). The transferred membrane was incubated firstly with 2 % bovine serum albumin (BSA) in phosphate buffered saline/Tween 20 (PBST) for one hour, followed by primary antibody (Anti-β-IFN Mouse mAb, Calbiochem, Merck Millipore, Darmstadt, Germany) in 1 % BSA/PBST for one hour and then with secondary antibody (Goat Anti-Mouse Total Ig Peroxidase conjugate, Calbiochem, Merck Millipore, Darmstadt, Germany) in 1 % BSA/PBST for one hour. The membrane was washed with PBST for three times after each incubation period. The blot was then developed using diaminobenzidine (DAB) tetrahydrochloride and hydrogen peroxide.

*Dot blot*

1.5 μL of the extracted recombinant hINF-β was applied to a nitrocellulose membrane and allowed to dry. The membrane was incubated for one hour at 4 °C in a blocking solution consisting of 2 % BSA in TBS-T pH 7.5. The membranes were washed three times for 10 min with TBS-T and incubated for one hour with Anti-β-IFN Mouse mAb (Calbiochem, Merck Millipore, Darmstadt, Germany) diluted 1:500 in TBS-T. The membranes were washed three times for 10 min with TBS-T and then, after addition of the secondary antibody, the rhINF-β spots were revealed. After color development, the membranes were washed out with water and the color changing was evaluated in both transformed and wild strains.

## Results

*Construction of the expression vector*

**Figure 1:**
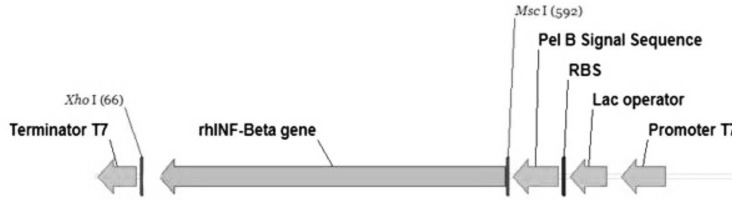
Schematic map of the designed plasmid showing the arrangement and composition of the synthetic cassettes inserted into pET-25b(+).The unique restriction sites inserted into the designed plasmid are *Msc*I and *Xho*I

**Figure 2:**
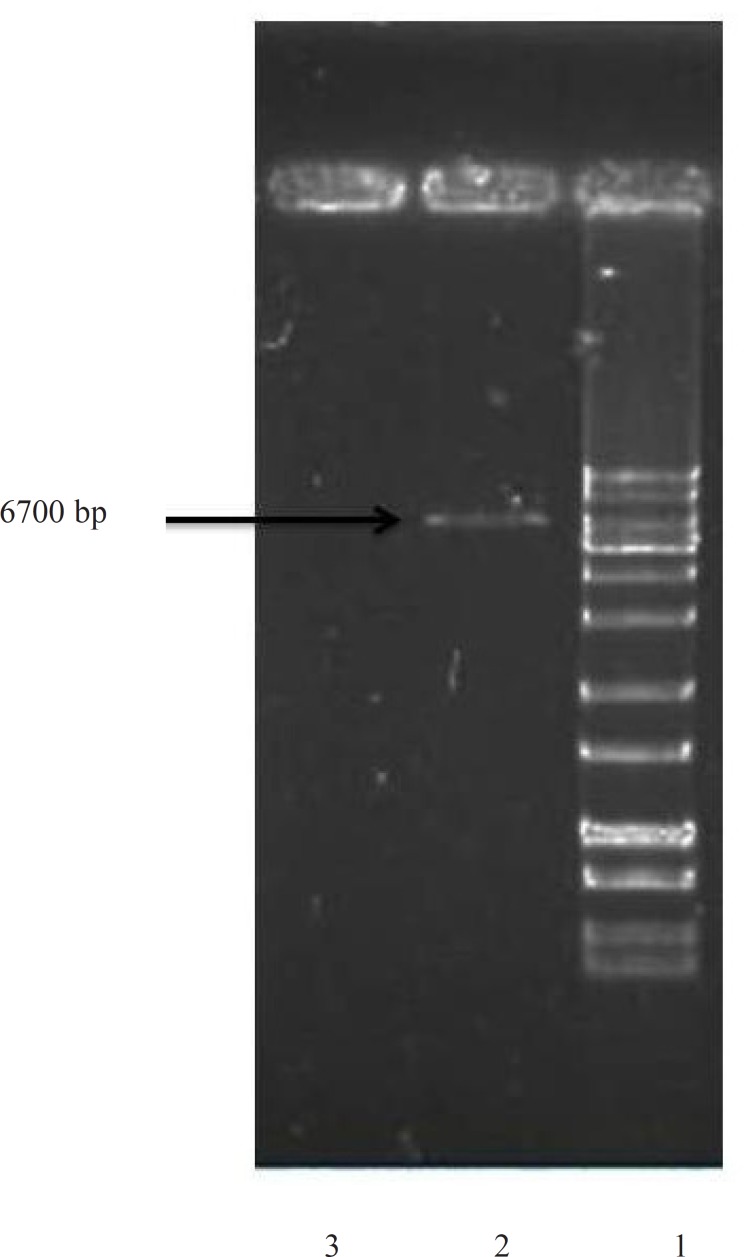
Confirmation of rhINF-β encoding gene in *Escherichia coli *after plasmid extraction by 0.8 % agarose gel electrophoresis. Lane 1: GeneRuler™ 1 kb DNA Ladder (Fermentas), Lane 2: *Escherichia coli *BL21 (DE3) with plasmid (Positive control), Lane 3: *Escherichia coli *BL21 (DE3) without plasmid (Negative control).

For an efficient expression of rhINF-β in *Escherichia coli*, a codon optimized construct was synthetized according to the optimal codon usage for *Escherichia coli *with GC content adjustment. Cysteine 17 was also replaced with serine to avoid intramolecular disulfide bridges and multimer formation. The designed construct was analyzed using VectorNTI 11.0 to ensure that the start codon and ribosome binding site of the mRNA were not in the stem and the construct is suitable for expression. There were no repeat sequences (including direct repeats, reverse repeat, and dyad repeats), premature polyA sites, CpG islands, internal Chi sites or cryptic splicing sites in the designed plasmid. The codon usage bias for the prokaryotic host, the GC content and the CpG dinucleotides content of the sequence was regarded to ensure efficient overexpression of the rhINF-β. Moreover, the mRNA secondary structure analysis was performed to find the potential RNA instability motifs or AU-rich elements (AREs) which determine the RNA stability. Restriction analyses showed no restriction sites that may interfere with cloning procedure. The plasmid pET-25b (+) was used as a template to construct vectors that target rhINF-β to the *E. coli *periplasm. The plasmid contains a T7 promoter, Lac operon, N-terminal *pelB *signal sequence for periplasmic localization, multiple cloning sites, rhINF-β encoding gene and T7 terminator. The schematic map of the designed plasmid is shown in Figure 1. After extraction, the recombinant plasmid was verified by 0.8 % agarose gel electrophoresis analysis (Figure 2).

*Growth profile of wild and recombinant strains*

**Figure 3:**
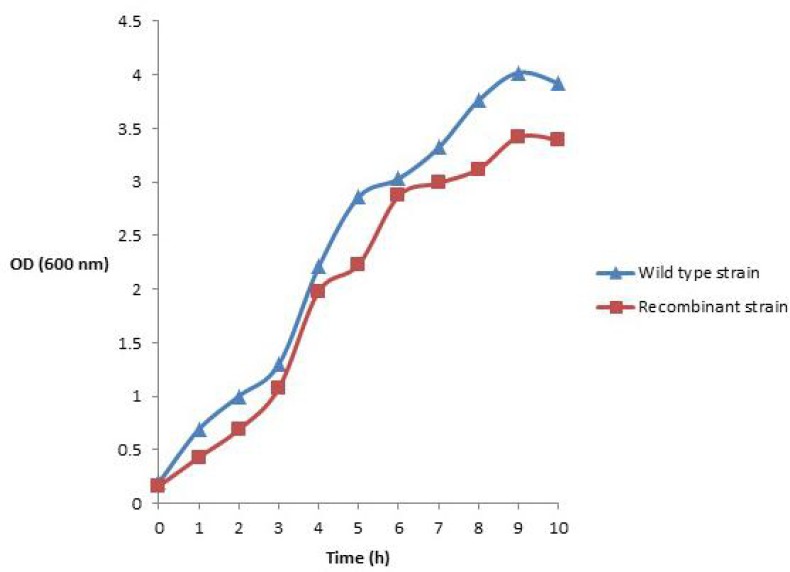
Growth profile, measured as optical density (OD) at 600 nm, of (a**) **wild type and (b) recombinant *E. coli *BL21 (DE3) cells for 10 h in batch fermentation experiment. Each experiment was performed in duplicate and the mean values were used to draw the plot

The effects of plasmid transformation and periplasmic recombinant hINF-β expression on *E. coli *BL21 (DE3) growth profile in shake flask and bioreactor are shown in Figure 3. It could be seen that the wild type cells entered the exponential phase before the rhINF-β expressing cells. The wild type cells showed the higher amount of cell growth, while the rhINF-β expressing cells represented a lower cell growth.

*Expression analysis of the recombinant hINF-β*

**Figure 4:**
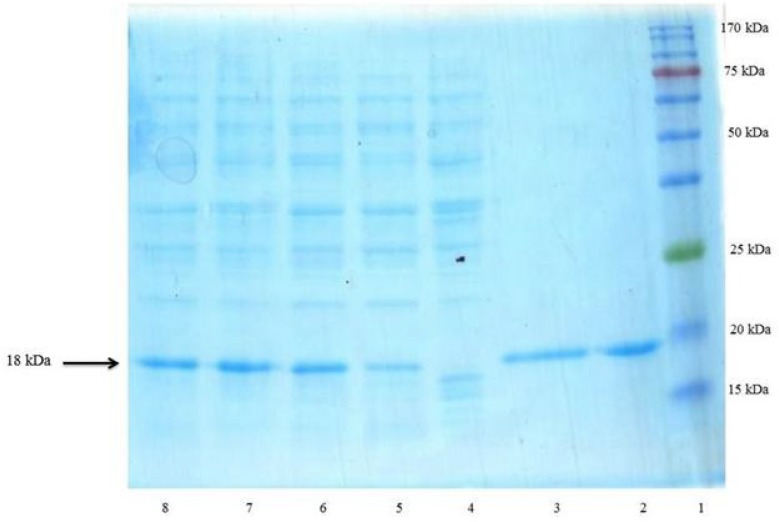
SDS–PAGE analysis of rhINF-β periplasmic expression in *E. coli*. Lane 1: Chromatine prestained protein ladder (SinaClon, Tehran, Iran), Lane 2: rhINF-β as positive control (ZiferonTM, Zist Daru Danesh, Tehran, Iran), Lane 3: rhINF-β as positive control (Betaseron®, Bayer HealthCare, Germany), Cell lysates were analyzed before IPTG addition (Lane 4) and 1-4 h after addition of 0.2 mM IPTG (Lanes 5-8). The additional band with molecular weight of 18 kDa in periplasmic fraction after induction corresponds to rhINF-β. The arrow indicates position of rhINF-β

The prepared vector encoding rhINF-β was transformed into the host cells for recombinant protein production. The expression of the recombinant protein was induced using IPTG at final concentrations of 0.2 mM in a 5 L bench top bioreactor at 37 °C. IPTG addition induces the expression of T7 RNA polymerase resulting in transcription of the rhINF-β gene under the control of the T7 promoter in the recombinant cells harboring the constructed plasmid. The N-terminal *pelB *secretion signal targets the translated protein in its unfolded state to the *E. coli *periplasm via the Sec-dependent transport pathway (31). The signal peptide is cleaved and the recombinant protein folds in periplasm with the help of chaperones and disulfide bond 

isomerases. Following the induction, the total periplasmic protein of induced and non-induced cells was compared on a SDS-polyacrylamide gel (Figure 4). The presence of corresponding protein bands confirmed the expression of the rhINF-β.

**Figure 5:**
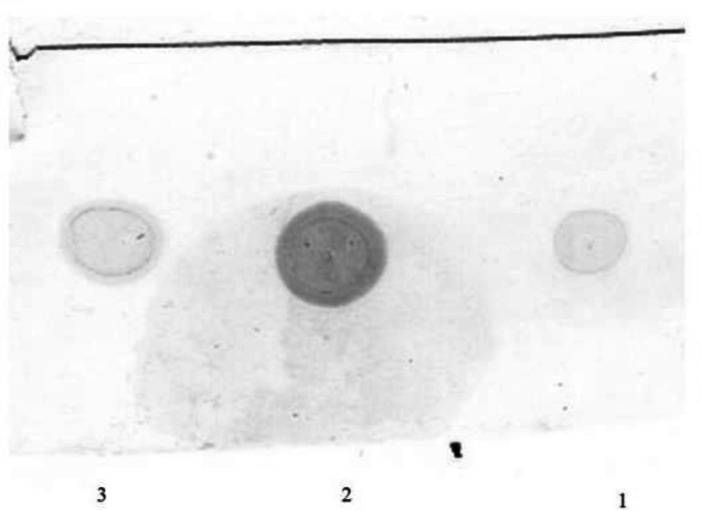
Dot blot analysis of the extracted periplasmic proteins. Lane 1: Reference standard (Betaseron® Bayer HealthCare, Germany), Lane 2: The extracted proteins from periplasmic space of the recombinant host containing the rhINF-β, Lane 3: Reference standard (ZiferonTM, Zist Daru Danesh, Tehran, Iran).

The total extracted periplasmic proteins from both recombinant and wild strain were used for dot blot assay (Figure 5). A signal was observed in dot blot analysis of bacterial lysate using antiserum against rhINF-β which confirms gene expression in the recombinant *Escherichia coli *BL21 (DE3) strain. 

**Figure 6:**
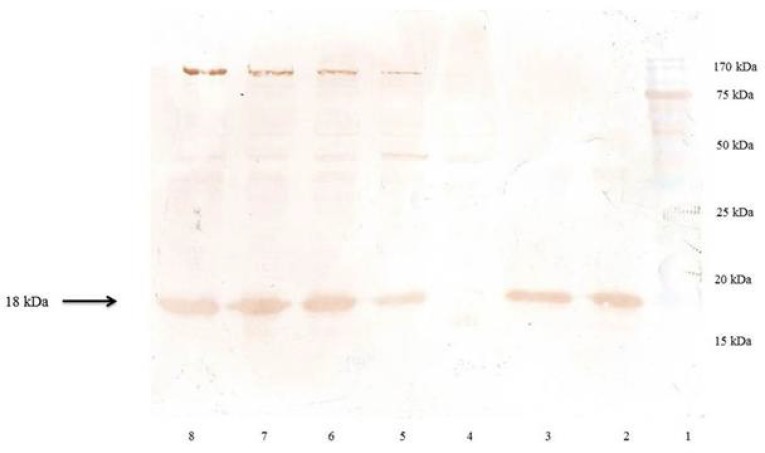
Western blot analysis of the rhINF-β. Lane 1: Chromatine prestained protein ladder (SinaClon, Tehran, Iran), Lane 2: rhINF-β as positive control (ZiferonTM, Zist Daru Danesh, Tehran, Iran), Lane 3: rhINF-β as positive control (Betaseron®, Bayer HealthCare, Germany), Cell lysates were analyzed before IPTG addition (Lane 4) and 1-4 h after addition of 0.2 mM IPTG (Lanes 5-8). The additional band with molecular weight of 18 kDa in periplasmic fraction after induction corresponds to rhINF-β. The arrow indicates position of rhINF-β

The produced protein was reactive to rabbit-anti-hINF-β antibody as evidenced by western blot analysis (Figure 6). The molecular weight of the recombinant protein is in accordance with the calculated molecular weight of recombinant hINF-β (~18 kDa). 

The rhINF-β represents approximately 35 % of the total cell protein according to the densitometry analysis. The results of SDS-PAGE, dot and western blot analysis confirmed the successful secretion of the processed protein into the periplasm.

Regarding the total protein content of the recombinant strain using Bradford method, the amount of the pure produced protein after SDS-PAGE analysis was measured by gel densitometry and found to be about 0.32 g L^-1^of culture medium.

## Discussion

*Escherichia coli *have been used as a heterologous expression host for both industrial and academic research applications. The periplasmic space of this Gram negative bacterium is of particular interest for functional production of a variety of recombinant proteins specially those which show toxic effects on host cells, or need the oxidizing environment of periplasm for appropriate formation of the structural disulfide bonds. Thus, in this study, periplasmic secretion strategy has been used for expression of rhINF-β. 

A new pET based broad host range expression plasmid for inducible production of rhINF-β in the periplasm of the Gram negative host *E. coli*. The designed vector was equipped with a set of sequences which enabled rhINF-β production in the bacterial periplasm following Sec-mediated export.

The results showed that the growth rate of *E. coli *cells harboring the recombinant plasmid is affected. A number of possible host-plasmid interactions which lowers the growth rate and biomass content of recombinant cells are described. It has been supposed that the expression level of plasmid-encoded proteins, replication and maintenance of the plasmid and disruption of cellular regulatory status (32), may inhibit the recombinant cells to reach to their maximum growth rate and biomass content. On the other hand, the cell growth was not affected severely. This shows that the rhIFN-β expressed by these strain did not exert toxicity on the cells during the fermentation.

Considering the fact that the periplasm is maximum 40 % of the total cellular volume (33), the protein yield (35 %) is considered to be acceptable and productive because of the ease and low costs of scaling up the procedure. Ghane *et al. *(34) have applied a synthetic gene in the T7 promoter/RNA polymerase system for rhIFN-β production to achieve a 28 % of the total cell protein. In another study, Krishna Rao *et al. *(20) have established a process for rhIFN-β to yield 34 % of the total cellular proteins. Whilst, in this study we obtained up to 35 % of the total cell protein which is the highest reported yield so far. For production purposes it might prove advantageous to generate microorganisms which secrete the desired protein into the medium. This strategy specially could be useful in recombinant production of some proteins like beta interferon which shows toxic effects on its host strain.

A number of signal sequences have been employed for efficient secretory production of heterologous proteins in *E. coli *via the Sec-dependent secretion pathway, including pectate lyase B (*pelB*), alkaline phosphatase (*phoA*), outer membrane protein A (*ompA*), and heat-stable enterotoxin 2 (*StII*) (35). The data indicate that *pelB *is an appropriate choice which can effectively be used for accumulation of the foreign produced proteins in the periplasmic space of *E. coli*.

## Conclusion

In conclusion, application of a vector engineering strategy was successful to improve rhINF-β production. This enhancement of production yield by periplasmic expression of the recombinant proteins suggests that this strategy may be successfully applied to practical large-scale high cell density fermentations of *E. coli *expression systems. The efficient host-vector systems are still a major challenge in pharmaceutical biotechnology. The cost-effective and economically feasible process developed in this study could be applicable in the development of other suitable therapeutic proteins expression in *E. coli*.
